# Transformed *Salmonella typhimurium* SL7207/pcDNA-*CCOL2A1* as an orally administered DNA vaccine

**DOI:** 10.1186/s13568-023-01650-8

**Published:** 2024-01-09

**Authors:** Juan Long, Yang Zeng, Fei Liang, Nan Liu, Yongzhi Xi, Yuying Sun, Xiao Zhao

**Affiliations:** Department of Immunology and National Center for Biomedicine Analysis, Senior Department of Hematology, Fifth Medical Center of Chinese PLA General Hospital, No.8, Dongda Ave, Fengtai District, Beijing, 100071 China

**Keywords:** Attenuated *Salmonella typhimurium* SL7207, DNA vaccine, Oral immune, Stability, Transformed strain SL7207/pcDNA-*CCOL2A1*

## Abstract

The use of attenuated bacteria for oral delivery of DNA vaccines is a recent innovation. We designed and constructed the naked plasmid DNA vaccine pcDNA-*CCOL2A1*, which effectively prevented and treated a rheumatoid arthritis model by inducing immunotolerance. We aimed to ensure a reliable, controllable dosage of this oral DNA vaccine preparation and establish its stability. We transformed pcDNA-*CCOL2A1* via electroporation into attenuated *Salmonella typhimurium* SL7207. A resistant plate assay confirmed the successful construction of the transformed strain of the SL7207/pcDNA-*CCOL2A1* oral DNA vaccine. We verified its identification and stability in vitro and in vivo. Significant differences were observed in the characteristics of the transformed and blank SL7207 strains. No electrophoretic restriction patterns or direct sequencing signals were observed in the original extract of the transformed strain. However, target gene bands and sequence signals were successfully detected after PCR amplification. *CCOL2A1* expression was detected in the ilea of BALB/c mice that were orally administered SL7207/pcDNA-*CCOL2A1*. The pcDNA-*CCOL2A1* plasmid of the transformed strain was retained under the resistant condition, and the transformed strain remained stable at 4 °C for 100 days. The concentration of the strain harboring the pcDNA-*CCOL2A1* plasmid was stable at 10^9^ CFU/mL after 6–8 h of incubation. The results demonstrated that the transformed strain SL7207/pcDNA-*CCOL2A1* can be expressed in vivo, has good stability, and may be used to prepare the oral DNA vaccine pcDNA-*CCOL2A1* with a stable, controllable dosage and the capacity to provide oral immunization. This vehicle can effectively combine both oral immunotolerance and DNA vaccination.

## Key points

- Attenuated *Salmonella typhimurium* SL7207 aids immunization as a DNA vaccine carrier.
- The transformed strain SL7207/pcDNA-*CCOL2A1* was constructed as an oral DNA vaccine.
- The formulated oral DNA vaccine offers a reliable, stable, and adjustable dosage.

## Introduction

Immunization with traditional DNA vaccines is administered mainly via intramuscular or subcutaneous injection, and gene gun. Although oral immunization is also practiced, its efficacy may be diminished by gastrointestinal digestion and degradation. In recent years, a novel vaccine strategy has been proposed wherein attenuated bacteria are used as oral carriers that deliver DNA vaccines to the host immune system (Lin et al. 2015; Yurina 2018). Bacterial replication may be partially impeded by gene mutation, and intracellular invasive bacteria are attenuated by repressing their principal virulence genes. The attenuated bacteria nonetheless retain their immunogenicity and can effectively stimulate the host immune response via oral administration without causing clinical disease. The efficacy and safety of this modality have been validated in several preclinical and clinical trials (Lin et al. 2015). Attenuated *Salmonella typhimurium* is an intestinal bacterium. Notably, various attenuated strains have been constructed via gene mutation. Among these, SL7207 is a *Salmonella* variant deficient in *aroA*, which encodes branching acid synthesis growth factor. This growth factor is crucial for the aromatic amino acid synthesis and metabolism pathways. Mutants with this gene deletion cannot be replicated in vivo as they do not synthesize the aforementioned growth factor. Therefore, orally administered attenuated strain SL7207 is expected not to induce symptoms of bacterial infection (Berger et al. 2013; Cobb et al. 2021; Johnson et al. 2017; Wang et al. 2016). Hence, the use of attenuated *Salmonella typhimurium* SL7207 as a DNA vaccine carrier ensures carrier safety as well as oral immunization for the DNA vaccine.

The naked plasmid pcDNA3.1-*CCOL2A1* (Song et al. 2009; Zhao et al. 2019a, 2021) encodes heterologous type II collagen, which effectively prevented and treated a rheumatoid arthritis (RA) model. pcDNA3.1-*CCOL2A1* was transformed via electroporation into SL7207 to construct the transformed strain SL7207/pcDNA-*CCOL2A1*, which could be used to prepare fixed doses of SL7207/pcDNA-*CCOL2A1* oral DNA vaccine and achieve oral immunotolerance of an antigen-specific gene vaccine. Different dosages of this oral DNA vaccine have been administered by gavage and shown prophylactic or therapeutic efficacy against the RA model in our laboratory (data not published). The dosages of oral vaccines are usually calculated on the basis of the number of bacterial colony-forming units (CFU). The proportion of efficacious strain (in total CFU) that carries the targeted plasmid is directly related to the strength of the immune response induced by this preparation. To ensure a reliable, controllable dosage of this oral DNA vaccine preparation, we identified the transformed strain SL7207/pcDNA-*CCOL2A1* both in vitro and in vivo and established its stability.

## Materials and methods

### Construction of transformed strain SL7207/pcDNA-*CCOL2A1*

Frozen attenuated *Salmonella typhimurium* SL7207 (constructed using the original strain [Strain Number: BNCC108207] by Kunming Institute of Zoology, Chinese Academy of Sciences, Kunming, Yunnan, China) cell suspension was rapidly thawed in a water bath at 37 °C. A 500 µL suspension was then transferred to 2 mL freshly prepared Luria–Bertani (LB) medium (1% tryptone [Oxoid, Hampshire, United Kingdom], 1% NaCl [XL chemical, Guangdong, China], and 0.5% yeast extract [Oxoid]) at a pH of 7.0, incubated at 37 °C, and shaken at 220 rpm for 6 h. The suspension was then dipped in an inoculum ring, which was used to draw a line on the LB plate. The latter was incubated overnight at 37 °C. Single SL7207 colonies were randomly selected and cultured in 10 mL LB broth with shaking at 37 °C for 8 h. One mL single colony suspension was then transferred to 100 mL LB (1:100) and shaken at 37 °C for 6–8 h. The suspension was then centrifuged (Beckman Coulter, Brea, CA, USA) at 2450 × *g* and 4 °C for 10 min to collect the bacteria. Precooled deionized water (10 mL) was then used to resuspend the cells, and they were centrifuged at 2450 × *g* at 4 °C for 10 min. The supernatant was discarded. The preceding step was repeated twice to wash the cells thoroughly. Thereafter, 100 µL cells were suspended in 0.5 mL of 15% (w/v) glycerol (Sigma-Aldrich, St. Louis, MO, USA) and placed in 1 mL EP tubes (Eppendorf GmbH, Hamburg, Germany). The SL7207 competent cells were then stored at − 80 °C until later use.

The pcDNA-*CCOL2A1* plasmid (total size approximately 10 kb) constructed in our laboratory (Song et al. 2009; Zhao et al. 2019a, 2021) was transformed into the SL7207 competent cells via electroporation with an Eporator (Eppendorf GmbH). The electric conversion conditions were 1.7 kV (Panel 1) and automatic electric shock time adjustment by the instrument. One mL LB was then added immediately after electroporation, and the suspension was shaken at 37 °C for 45 min. Subsequently, 200 µL of the transformed strains was plated on LB containing 100 g/L ampicillin (Solarbio, Beijing, China) (LB (Amp +)) and incubated at 37 °C for 16 h.

### Identification of the transformed strain SL7207/pcDNA-*CCOL2A1 *in vitro

The following in vitro identification procedure was followed to confirm the successful construction of the transformed strain SL7207/pcDNA-*CCOL2A1*:

*Plate identification*. Single colonies that grew on the LB (Amp +) plate after electroporation were randomly selected and cultured in the LB (Amp +) solution at 37 °C. A 100 µL bacterial suspension harvested after gradient dilution (1:10^7^, by mixing 100 μL of the serial dilutions of the bacterial suspension with 900 μL of the LB solution) was then spread onto the LB (Amp +) plate and incubated at 37 °C. Colony growth was visually inspected after 16 h.

*Identification of characteristics*. Both transformed and blank SL7207 strains were cultured at an initial optical density of 600 nm (OD_600_) = 0.1 in 100 mL LB (Amp +) and LB (Amp −) solutions with shaking at 220 rpm and 37 °C for 6 h. Cell suspension OD_600_ was measured with a Synergy HT Multi Mode Microplate Reader (BioTek Instruments Inc., Winooski, VT, USA). Cell suspensions were also spread onto LB (Amp +) and LB (Amp − ) plates after gradient dilution (similar to the abovementioned method) and incubated at 37 °C for 16 h. Differences between the two strains in terms of cell suspensions and plate colonies were visually inspected.

*Identification using enzymatic digestion*. The plasmid DNA in the transformed strain SL7207 was extracted, purified with a Wizard^®^ Plus SV Minipreps DNA Purification System Kit (Promega, Madison, WI, USA), digested with *Eco*RI and *Hin*dIII restriction endonucleases (Takara, Dalian, China), and identified using electrophoresis (Juan et al. 2017). The positive control was plasmid DNA extracted from transformed strain *E. coli*/pcDNA-*CCOL2A1* via double restriction endonuclease digestion.

*Identification *via* sequencing*. The extract was subjected to first-generation Sanger sequencing (ABI 3730; Applied Biosystems Inc., Foster City, CA, USA) using the universal primers CMV-F and BGH-R of pcDNA3.1. The extract was then sequenced after full-length PCR amplification using an approximately 4-kb fragment size. An Applied Biosystems 9700 PCR System (Applied Biosystems Inc.) was used under the following conditions: initial denaturation at 98 °C for 3 min, followed by 30 cycles of denaturation at 98 °C for 10 s, annealing at 65 °C for 10 s, and extension at 72 °C for 65 s, and final extension at 72 °C for 5 min. Full-length PCR amplification was performed using Phanta™ Super-Fidelity DNA polymerase (Vazyme Biotech Co. Ltd., Piscataway, NJ, USA) in 50-µL reaction systems. The amplification primers are listed in Table 1. The harvested PCR stock solution was subjected to *CCOL2A1* sequencing analysis, and the primers were designed according to the gene sequence. The extract was rapidly identified by sequencing the partial PCR amplification using the amplification primers listed in Table 1. The amplified fragment was 1928 bp in size, and the size range was 1159–3086 bp. The volume of the amplification system was 50 µL, and the amplification conditions were as follows: initial denaturation at 98 °C for 3 min, followed by 30 cycles of denaturation at 98 °C for 10 s, annealing at 59.9 °C for 10 s, and extension at 72 °C for 40 s, and final extension at 72 °C for 5 min. The amplified products were analyzed through 1% agarose gel electrophoresis at 150 V and 60 A for 40 min. The remaining PCR stock sample was then sequenced using the amplification primers listed in Table 1. All sequences were compared against those reported with DNAMAN v. 5.2.2 (Lynnon Biosoft Corp., San Ramon, CA, USA).

**Table 1:** Amplified fragment size and full-length^a^/partial sequence primers from the SL7207/pcDNA-*CCOL2A1* extract determined using PCR

Primer	Sequence (5ʹ to 3ʹ)	Size (bp)
Full-length amplified forward primer	GATCAAGCTTGTTATGGATATGCATGGACGTC	~ 4000
Full-length amplified reverse primer	GATCGAATTCTTACAAGAAGCAGACTGGGCC	
Partial amplified forward primer	CTGGGACCCAAAGGACAGAC	1928
Partial amplified reverse primer	GTCTCGCCTCTGTCTCCTTG	

^a^‘GATC’ at the start of a full-length amplified primer is the protective base

### Identification of the transformed strain SL7207/pcDNA-*CCOL2A1 *in vivo

BALB/c mice aged 4–5 weeks were purchased from Beijing Vital River Laboratory Animal Technology Co. Ltd. (Beijing, China), raised under China National Accreditation Service-accredited specific pathogen-free conditions, and maintained in accordance with the ARRIVE Guidelines for the Care and Use of Laboratory Animals. All work was approved by the Chinese People’s Liberation Army General Hospital Animal Welfare Committee.

Five male and five female mice were inoculated by intragastric gavage administration with transformed strain SL7207/pcDNA-*CCOL2A1* at a dose of 10^9^ CFU/animal. The mice were fasted for 12 h, deprived of water for 4 h, and intragastrically administered 0.1 mL of 10% (w/v) sterilized NaHCO_3_ to neutralize stomach acid 30 min before SL7207/pcDNA-*CCOL2A1* administration. The blank strain SL7207 was administered as a negative control. On day 3 after inoculation, the terminal ilea with their concentrated Peyer’s nodes were collected and pulverized in liquid nitrogen. Total RNA was extracted and reverse-transcribed using RT-qPCR to detect *CCOL2A1* mRNA in the ileal tissue. *β*-actin was used as the internal reference for relative quantitative analysis. The probes, primers, and amplified fragment sizes used in the RT-qPCR are listed in Table 2.

**Table 2:** Amplified fragment size, primer, and probe sequences of *CCOL2A1* and *β*-actin determined using qPCR from in vivo expression identification

Gene	Primer/Probe	Sequence (5′ to 3′)	Size (bp)
*β-actin*	Mouse *β-actin* F-primer	ACCGTGAAAAGATGACCCAGAT	70
	Mouse *β-actin* R-primer	GCCTGGATGGCTACGTACATG	
	Mouse *β-actin* probe	(FAM)TTTGAGACCTTCAACACC(MGB)	
*CCOL2A1*	*CCOL2A1* F-primer	TCTTGTTGGTCCCAGAGGTGA	118
	*CCOL2A1* R-primer	ACCCTTGGGTCCGTCAGTG	
	*CCOL2A1* probe	(FAM) CGTGGATTCCCCGGTGAACGC(MGB)	

### Stability of the transformed strain SL7207/pcDNA-*CCOL2A1*

The genetic stability of the transformed plasmid in the SL7207 cells was verified using two similar methods.

*Method 1*. The plasmid retention rate was determined by referring to the “231 plasmid loss rate test method” under the bioassay item in the Fourth General Rule of Chinese Pharmacopoeia (2020 edition). The positively transformed strains were cultured in LB (Amp +) solution, serially diluted, spread onto nonselective LB (Amp −) plates, and cultured overnight at 37 °C. At least 100 well-growing single colonies were then randomly selected and spread onto replicate LB (Amp − ) and LB (Amp +) plates. The ratio of the number of CFU on a selective plate to that on a corresponding nonselective plate was used to calculate the plasmid retention rate (n = 2), indicating the proportion of plasmid-bearing bacterial cells.

*Method 2*. The positively transformed strains were inoculated in LB (Amp +) solution. Equal volumes of transformants were serially diluted, spread onto nonselective LB (Amp −) and selective LB (Amp +) plates, respectively, and cultured overnight at 37 °C. The proportion of plasmid-bearing bacterial cells was determined by calculating the ratio of the number of CFU on the LB (Amp +) plate to that on the LB (Amp −) plate. The percentage of cells in the population retaining the recombinant plasmid indicated the genetic stability of the transformed strain SL7207/pcDNA-*CCOL2A1*.

The stability of the transformed strain SL7207/pcDNA-*CCOL2A1* was also evaluated during 22 h continuous culture. The transformed strain SL7207/pcDNA-*CCOL2A1* was cultured at initial OD_600_ = 0.1 in 100 mL LB (Amp +) solution with shaking at 220 rpm and 37 °C for 22 h. Samples were collected every 2 h, and the OD_600_, CFU, and plasmid retention rate were measured for each sample. Six- and eight-hour cultures were serially conducted to establish the optimal incubation time and ensure the stability of the transformed strain SL7207/pcDNA-*CCOL2A1*.

The stability of the transformed strain SL7207/pcDNA-*CCOL2A1* over 100 days of storage at 4 °C was also assessed. A bacterial suspension derived from a single colony stored at 4 °C was used to prepare oral vaccines (SL7207/pcDNA-*CCOL2A1*) on different days. The CFU and plasmid stability of the preparations were determined to evaluate the stability of the transformed strain SL7207/pcDNA-*CCOL2A1* during long-term storage at 4 °C.

### Statistical analysis

Data were analyzed with SPSS v. 13.0 (SPSS Inc., Chicago, IL, USA). For the in vivo experiments, differences in relative expression were examined using the Kruskal–Wallis H test for multiple samples. The threshold for statistical significance was defined as P < 0.05.

## Results

### Transformed strain SL7207/pcDNA-*CCOL2A1* was successfully constructed

After 16 h incubation at 37 °C, numerous signal colonies appeared on the LB (Amp +) plate. Hence, the target plasmid was successfully electroporated into SL7207, and the transformed strain SL7207/pcDNA-*CCOL2A1* was successfully constructed.

### Characteristic differences between the transformed and blank strains

Under the same culture conditions, the two bacterial suspensions markedly differed in terms of appearance, OD_600_, post-centrifugation properties of the upper liquid, natural bacterial sedimentation characteristics, and colony size and morphology (Table 3).

**Table 3:** Differences in the characteristics of transformed and blank SL7207 strains

Strain	Appearance	OD_600_	Upper liquid	Sedimentation characteristics	Colony size	Colony morphology
SL7207	High turbidity	> 3.50 (3.68–4.50)	Turbid	Slow settling, white powdery sediment, rapid dispersal after shaking	Relatively small	Compact, whitish
SL7207/pcDNA-*CCOL2A1*	Lower turbidity than SL7207	< 3.00 (2.17–2.95)	Clear	Fast settling, viscous ribbon sediment, difficult dispersal, and persistent ribbon flocs after shaking	Relatively large	Mellow, translucent

### Electrophoresis revealed no target bands after enzymatic digestion

**Fig. 1:**
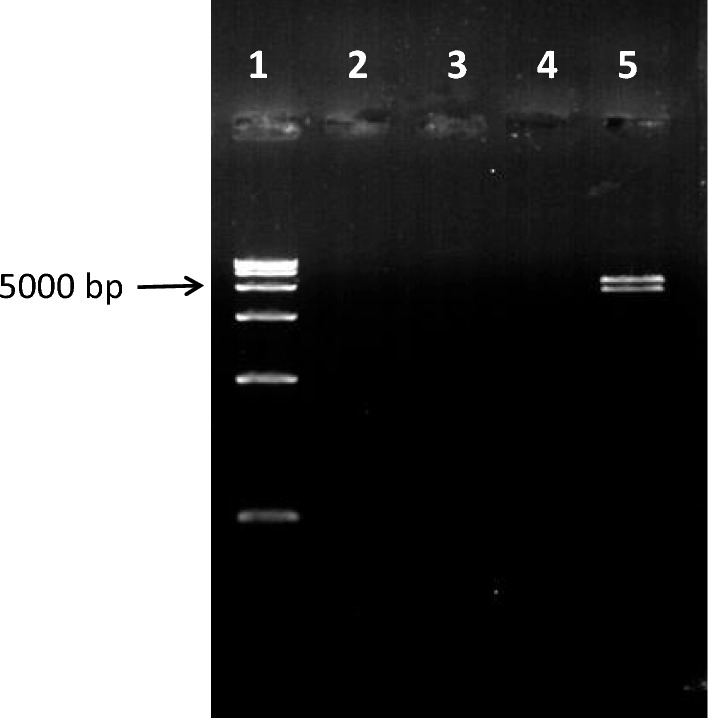
Electrophoresis restriction patterns. 1. Marker (DNA Ladder [D15000]; TIANGEN, Beijing, China). 2. Original extract of the transformed strain SL7207. 3. Product of the extract obtained from the transformed strain SL7207 using *Eco*RI restriction enzyme*.* 4. Product of the extract obtained from the transformed strain SL7207 using *Eco*RI and *Hin*dIII restriction enzymes. 5. Positive control (product of the extract obtained from the *E. coli*/pcDNA-*CCOL2A1* transformed strain using *Eco*RI and *Hin*dIII restriction enzymes)

Electrophoresis of the extract subjected to enzymatic digestion (Fig. 1) showed no bands on the original extract or the single- or double-enzyme digestion products. However, electrophoresis revealed double bands on the double-enzyme digestion product of *E. coli*/pcDNA-*CCOL2A1*. The target and vector bands were approximately 4,000 and 5,000 bp, respectively.

### Target signals in the post-PCR amplification sequencing

**Fig. 2:**
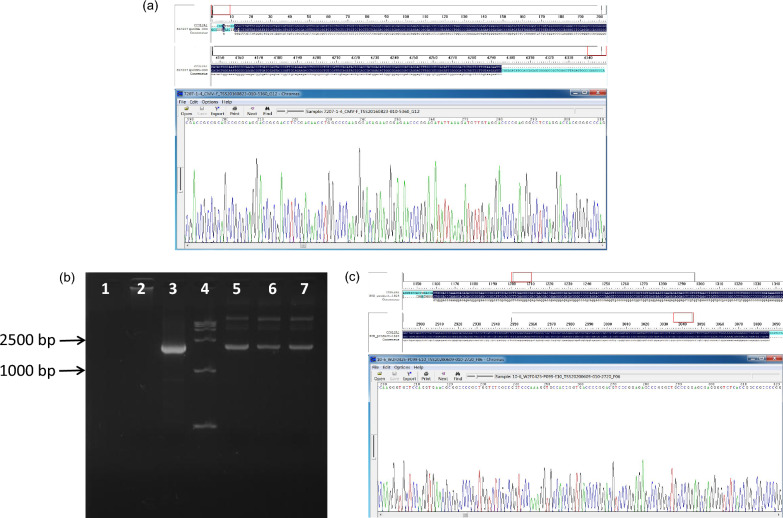
Identification of PCR amplification products via sequencing. **a** Representative sequence alignment (top/middle) and sequencing peak (bottom) diagrams of the amplification product from the full-length sequence using PCR. **b** Electrophoretic identification of the amplification product from a partial sequence using PCR. 1. Blank control, ddH_2_O template. 2: Negative control, original extract of blank strain SL7207 as template. 3: Positive control, pcDNA-*CCOL2A1* plasmid as template. 4. Marker. 5, 6, and 7. Amplification products using extracts of three single colonies from transformed SL7207 strains as templates. **c** Representative sequence alignment (top/middle) and sequencing peak (bottom) diagrams of the amplification product from a partial sequence using PCR. The pictures at the top, middle, and bottom in **a** and **c** are partially displayed

Direct BGH-R primer sequencing of the extract from the transformed strain SL7207 demonstrated no signal. Moreover, direct CMV-F primer sequencing generated a signal that was neither the target gene sequence nor the vector sequence. After full-length PCR amplification of the extract, sequencing generated a signal that was consistent with the target gene (Fig. 2a). After partial PCR amplification of the extract, the corresponding bands were detected via electrophoresis ((Fig. 2b)), and the signal was consistent with the target gene sequence (Fig. 2c). Thus, electrophoresis and sequencing of the extract after PCR amplification indicated that the transformed strain SL7207 contained the plasmid carrying the target gene.

### Target gene in the transformed strain SL7207 could be expressed after oral administration in vivo

**Fig. 3:**
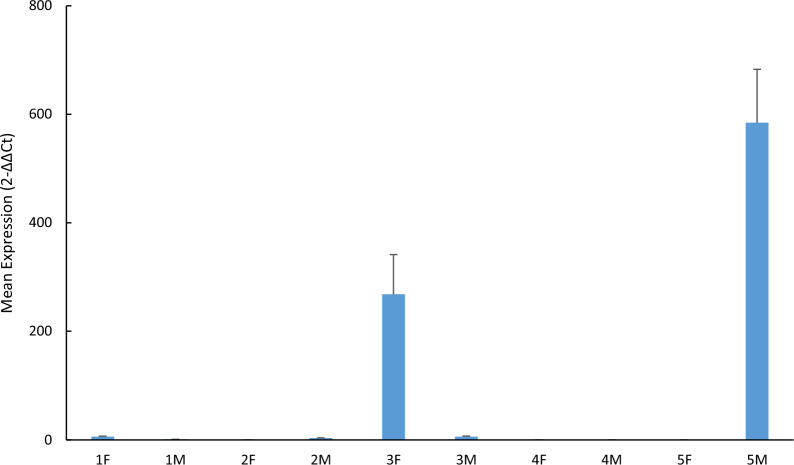
*CCOL2A1* expression in BALB/c mouse ilea. Bacterial suspension of the transformed strain SL7207/pcDNA-*CCOL2A1* (10^10^ CFU/mL) was administered by gavage at 4.0 × 10^9^ CFU and 0.4 mL per mouse. M: Male. F: Female. 1 M represents male mouse No. 1, 1F represents female mouse No. 1, and so on. Standards and samples for RT-qPCR determination were run in triplicate. Differences in relative expression were evaluated using the Kruskal–Wallis H test for multiple samples. χ^2^ (H statistic) = 16.111, df = 5, P = 0.007. Significant differences between samples indicated individual differences in *CCOL2A1* expression among mouse ilea. df: degree of freedom

Five male and five female BALB/c mice were administered the transformed strain SL7207/pcDNA-*CCOL2A1* by intragastric gavage at 10^9^ CFU/animal. On day 3 after treatment, the *CCOL2A1*-mRNA transcribed in the ileum was determined using RT-qPCR. The value measured for male mouse No. 1 (hereafter, 1 M) was used as the normalization reference for relative quantification (Fig. 3). Target gene expression was detected in six of the ten samples. The target gene expression levels were significantly (P < 0.05) higher in female mouse No. 3 (hereafter, 3F) and male mouse No. 5 (hereafter, 5 M) than all others. No amplified signals were detected in the negative or blank control (ddH_2_O template) samples. The mean Ct was 12.67 ± 0.22 for the positive control sample (pcDNA-*CCOL2A1* plasmid template). Thus, the RT-qPCR met the QC requirements.

### Transformed strain SL7207/pcDNA-*CCOL2A1* was stable and controllable within 6–8 h culture time

**Fig. 4:**
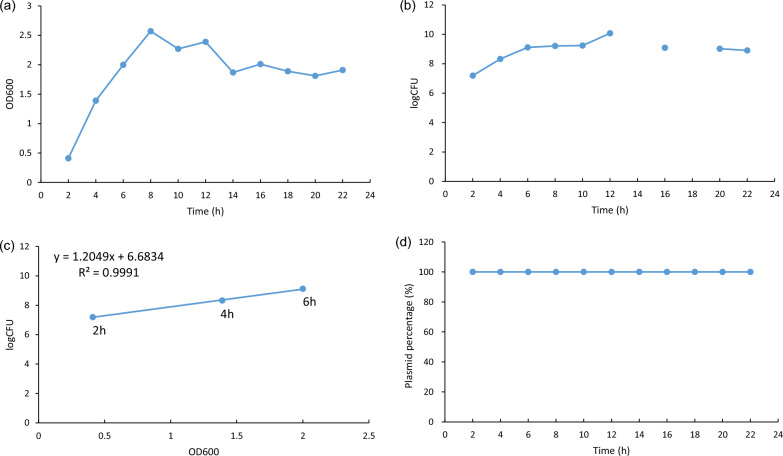
Stability of transformed strain SL7207/pcDNA-*CCOL2A1* during a 22-h continuous culture. **a** Cell growth curve at OD_600_. **b** Log CFU of bacterial broth. **c** Correlation between bacterial log CFU and OD_600_. **d** Plasmid retention rate. CFU could not be determined at 14 or 18 h in **b** because of irregular plate colony counts following gradient dilution

The transformed strain SL7207/pcDNA-*CCOL2A1* was incubated at 37 °C for 22 h. During that time, its growth curve, cell suspension CFU, and plasmid retention rate were measured (Fig. 4). The bacterial suspension OD_600_ proportionately increased over 8 h of incubation (Fig. 4a). During incubation, the cell suspension concentration was stable at 10^9^ CFU/mL between 6 and 10 h but reached 10^10^ CFU/mL by 12 h (Fig. 4b). A positive linear correlation was observed between the log CFU and OD_600_ values during 6 h incubation (R^2^ = 0.9991; Fig. 4c). The plasmid retention rate of the transformed strain was 100% at every sampling point during continuous culture (Fig. 4d). The transformed strain SL7207/pcDNA-*CCOL2A1* was relatively stable, and the OD_600_ reflected the magnitude of the CFU over 6–8 h incubation under this culture condition. Repeated culture tests (Table 4) showed that the OD_600_ of the bacterial suspension harvested at 6 or 8 h incubation was relatively stable at 2.47 ± 0.13, the bacterial suspension was stable at 10^9^ CFU/mL, and the stability of the plasmid (Method 2) in the transformed strain was ≥ 90% (range, 90–120%). Therefore, the transformed strain was stable over 6 or 8 h incubation. Based on the controllability of the CFU, the stability of the plasmid, and the convenience of the sample preparation, the optimal culture time range was 6–8 h for the preparation of oral DNA vaccine from the transformed strain SL7207/pcDNA-*CCOL2A1*.

**Table 4:** Stability and productivity of the transformed strain SL7207/pcDNA-*CCOL2A1* after repeated shake flask culture for 6 and 8 h

Time (h)	OD_600_	CFU/mL (× 10^9^)	Plasmid stability (%)
6 h	2.52	2.07	99.04
8 h	2.28	1.63	93.13
6 h	2.36	1.24	96.87
8 h	2.44	1.49	117.28
6 h	2.71	1.49	91.80
8 h	2.47	1.84	105.74
6 h	2.52	2.38	113.72
8 h	2.47	1.23	97.35
*M* ± *SD*	2.47 ± 0.13	1.67 ± 0.40	101.87 ± 9.44

### Transformed strain SL7207/pcDNA-*CCOL2A1* was stable and controllable over the duration of 100 days at 4 °C

The stability of the transformed strain SL7207/pcDNA-*CCOL2A1* was evaluated by subjecting the bacterium to long-term storage at 4 °C. Table 5 shows that the concentration of the cell suspension derived from the same single colony and stored at 4 °C was stable at 10^9^ CFU/mL, and its plasmids were ≥ 90% stable at different sampling points over 100 days. Hence, the transformed strain SL7207/pcDNA-*CCOL2A1* was stable at 4 °C storage and could be used to prepare an oral DNA vaccine with a dosage that is controllable over the duration of 100 days.

**Table 5:** Stability of the transformed strain SL7207/pcDNA-*CCOL2A1* during long-term storage

Item	Storage time (days)									
	2 days	7 days	15 days	22 days	36 days	49 days	70 days	77 days	91 days	98 days
CFU/mL (× 10^9^)	2.65	2.59	1.44	1.49	1.66	2.35	1.51	1.11	1.83	1.53
Plasmid stability (%)	96.59	91.42	95.36	90.47	102.69	95.02	95.38	101.52	101.86	97.24

## Discussion

The naked plasmid pcDNA-*CCOL2A1* is an antigen-specific, immunotolerant DNA vaccine that demonstrated efficacy at preventing and treating RA models. The plasmid was directly transformed via electroporation into SL7207, and the exogenous gene harbored by the latter was orally administered to induce oral DNA vaccine immunotolerance (Berger et al. 2013; Hopkins et al. 2000; Garmory et al. 2003; Zheng et al. 2012). We identified and prepared the transformed strain SL7207/pcDNA-*CCOL2A1* for culture and in vitro and in vivo expression, respectively, and evaluated its stability. The oral DNA vaccine SL7207/pcDNA-*CCOL2A1* was successfully constructed, could be expressed in vivo, had good stability and controllable dosage, and was feasible for preparation and application.

Single colonies of the transformed strain SL7207/pcDNA-*CCOL2A1* harbored a recombinant plasmid bearing the ampicillin resistance gene and could be screened by adding a certain amount of ampicillin to the solid selection medium. After the positive transformants screened using the LB (Amp +) plate were cultured in LB (Amp +) solution, the appearance and characteristics of the resultant transformed strain markedly differed from those of the original blank strain. When the foreign plasmid was transformed into the SL7207 carrier, certain replicons in the plasmid structure domain altered the strain quality, density, and nature. Therefore, the transformants obviously differed in terms of their bacterial suspensions, cells, and colonies as well as their immunogenicity (Johnson et al. 2019; Wegrzyn and Wegrzyn 2002). However, the effects of the transformed strain SL7207 on the immune response and immunotolerance required further investigation.

To verify that the target plasmid was transformed into SL7207, the former was extracted from SL7207/pcDNA-*CCOL2A1* after 6 h culture, digested with restriction enzyme, and identified using electrophoresis and sequencing, following the identification method of *E. coli* plasmids (Juan et al. 2017). Nevertheless, electrophoresis disclosed no bands corresponding to the enzymatic digestion product, and sequencing revealed no target gene signal. However, a suitable density of single transformant colonies grew on LB (Amp +) plate. Furthermore, the transformant suspension from which the plasmid was extracted differed in appearance from the blank strain SL7207. The concentration of the effective strain was 10^9^ CFU/mL after 6 h incubation, and the plasmid stability was ≥ 90%. Therefore, the extract might have contained only a low concentration of the target gene-bearing plasmid. The microscale target gene in the extract was amplified using PCR to meet digestion and sequencing requirements, and the bands and signal generated were consistent with those of the target gene. Possibly, the plasmid purification kit currently available on the market can only extract target plasmids from transformed *E. coli* but not from transformed *Salmonella typhimurium*. The concentration of the extract was measured at OD_260_ and OD_280_ before enzymatic digestion and sequencing and was approximately 100 µg/mL. Moreover, A_260_/A_280_ was in the range of 1.8–2.0. However, the sequence alignment showed that the extract contained neither the target plasmid harboring the target gene nor the vector plasmid without the target gene. Therefore, extract concentration and purity alone cannot reliably confirm the identity of the transformed strain SL7207. Three methods may serve to identify transformed strain SL7207/pcDNA-*CCOL2A1 *in vitro: (1) observe the appearance of the bacterial suspension; (2) investigate single-colony growth through plate identification; and (3) perform electrophoresis and sequencing on low extract concentrations after PCR amplification. As PCR amplification and sequencing are cumbersome, they should only be performed for supplementary follow-up identification. Characteristics and plate identification are the fastest and most effective methods of screening transformed strain SL7207/pcDNA-*CCOL2A1* for the preparation of the oral DNA vaccine.

Despite individual differences in the in vivo* CCOL2A1* expression of the transformed strain SL7207/pcDNA-*CCOL2A1*, *CCOL2A1* was nonetheless detected in the ileal tissues after intragastric administration. Future research should endeavor to investigate the in vivo roles of the orally administered DNA vaccine. For example, the dynamic profiles and biodistribution of *CCOL2A1* should be investigated in peripheral blood and various tissues from normal animals or RA models (Zhao et al. 2019a). Serum anti-type II collagen (CII) IgG antibodies and ileum mucosal immune secretory immunoglobulin A (sIgA) should be examined to determine in vivo* CCOL2A1* expression (Jiang et al. 2017; Song et al. 2019; Zhao et al. 2019b, 2021).

The stability of the transformed strain SL7207/pcDNA-*CCOL2A1* must be established to ensure the stability and controllability of the oral DNA vaccine preparation. This analysis also helps understand the dynamic changes that occur in the target plasmid within the transformed strain, clarify the relationship between OD_600_ and CFU, and determine the optimal harvest time and CFU for the preparation of the oral DNA vaccine. The plasmid retention rate of SL7207/pcDNA-*CCOL2A1* was 100%, and the plasmid stability was ≥ 90% under current culture conditions. The OD_600_ reflected the CFU within a certain culture time. The foregoing findings indicate that the transformed strain SL7207/pcDNA-*CCOL2A1* was stable, and the stock strain could be used to prepare accurate doses of the oral preparations in LB (Amp +) solution within a certain time window. The stability of the strain, the controllability of the CFU, the convenience of preparation, and other factors can be integrated to determine the optimal shaking culture time and preparation method for the oral DNA vaccine derived from the aforementioned transformed strain. In this manner, subsequent research may be conducted on the clinical efficacy and safety and the molecular mechanism of the optimized oral DNA vaccine product. Disease incidence and progression, health status, and body weight have been monitored in intragastrically administered RA models to evaluate the anti-rheumatic efficacy and safety of the SL7207/pcDNA-*CCOL2A1* oral DNA vaccine. Serum rheumatoid-associated antibodies (such as anti-cyclic citrullinated peptide antibodies and rheumatoid factor), T lymphocyte subsets, and serum cytokines and chemokines have also been monitored to clarify immune system changes in our laboratory (data not published) (Kolarz et al. 2021; Malemud 2018; Kondo et al. 2021). Key genes and proteins should be screened through the high-throughput multi-omics joint analysis of the transcriptome, proteome, and metabolome (Chu et al. 2021; Zeng et al. 2022). The function and pathway mechanisms related to the prevention and treatment of RA need to be thoroughly explored in the gene–protein–cell axis to reveal the mechanism behind immunotolerance regulation of antigen-specific immunotherapy in RA.

In the present study, oral immunotolerance and DNA vaccination were combined to develop an antigen-specific, tolerizing oral DNA vaccine comprising the CII gene harbored by attenuated *Salmonella typhimurium* SL7207. The product was named SL7207/pcDNA-*CCOL2A1,* and its characteristics were closely associated with the bacterial gene carrier. Based on these results, a three-level in vitro identification standard for transformed strain SL7207 was established. The attenuated SL7207 could serve as a carrier to achieve in vivo DNA vaccine expression. The transformed strain SL7207/pcDNA-*CCOL2A1* with the “huge” plasmid pcDNA-*CCOL2A1* had good stability and was suitable for use in an oral preparation with an accurate dosage. Notably, achieving oral immunotolerance is crucial for treating autoimmune disease. Immunotolerance induced by allogeneic or heterogeneous specific antigens has become a hotspot in RA research as it does not induce generalized immunosuppression (Pozsgay et al. 2017; Burmester and Pope 2017; Abbasi et al. 2019; Mueller et al. 2021). The oral DNA vaccine developed herein could improve antigen stability and drug compliance, achieve oral immunotolerance to antigen-specific gene vaccines, and have high application value and scientific significance in this field.

## Data Availability

The datasets generated during the current study are available from the corresponding author on reasonable request.

## References

[CR1] Abbasi M, Mousavi MJ, Jamalzehi S, Alimohammadi R, Bezvan MH, Mohammadi H, Aslani S (2019). Strategies toward rheumatoid arthritis therapy; the old and the new. J Cell Physiol.

[CR2] Berger E, Soldati R, Huebener N, Hohn O, Stermann A, Durmus T, Lobitz S, Zenclussen AC, Christiansen H, Lode HN, Fest S (2013). Salmonella SL7207 application is the most effective DNA vaccine delivery method for successful tumor eradication in a murine model for neuroblastoma. Cancer Lett.

[CR3] Burmester GR, Pope JE (2017). Novel treatment strategies in rheumatoid arthritis. Lancet.

[CR4] Chu X, Zhang B, Koeken VACM, Gupta MK, Li Y (2021). Multi-omics approaches in immunological research. Front Immunol.

[CR5] Cobb J, Rawson J, Gonzalez N, Hensel M, Kandeel F, Husseiny MI (2021). Oral *Salmonella* msbB mutant as a carrier for a Salmonella-based vaccine for prevention and reversal of type 1 diabetes. Front Immunol.

[CR6] Garmory HS, Titball RW, Brown KA, Bennett AM (2003). Construction and evaluation of a eukaryotic expression plasmid for stable delivery using attenuated *Salmonella*. Microb Pathog.

[CR7] Hopkins SA, Niedergang F, Corthesy-Theulaz IE, Kraehenbuhl JP (2000). A recombinant *Salmonella typhimurium* vaccine strain is taken up and survives within murine Peyer’s patch dendritic cells. Cell Microbiol.

[CR8] Jiang H, Hu Y, Yang M, Liu H, Jiang G (2017). Enhanced immune response to a dual-promoter anti-caries DNA vaccine orally delivered by attenuated *Salmonella typhimurium*. Immunobiology.

[CR9] Johnson SA, Ormsby MJ, Wall DM (2017). Draft genome sequence of the tumor-targeting *Salmonella enterica* serovar typhimurium strain SL7207. Genome Announc.

[CR10] Johnson SA, Ormsby MJ, McIntosh A, Tait SWG, Blyth K, Wall DM (2019). Increasing the bactofection capacity of a mammalian expression vector by removal of the f1 ori. Cancer Gene Ther.

[CR11] Juan L, Xiao Z, Fang Y, Fei L, Nan L, Song Y, Ling W, Yuying S, Yongzhi X (2017). Genetic stability of an *Escherichia coli* strain engineered to produce a novel therapeutic DNA vaccine encoding chicken type II collagen for rheumatoid arthritis. Process Biochem.

[CR12] Kolarz B, Podgorska D, Podgorski R (2021). Insights of rheumatoid arthritis biomarkers. Biomarkers.

[CR13] Kondo N, Kuroda T, Kobayashi D (2021). Cytokine networks in the pathogenesis of rheumatoid arthritis. Int J Mol Sci.

[CR14] Lin IY, Van TT, Smooker PM (2015). Live-attenuated bacterial vectors: tools for vaccine and therapeutic agent delivery. Vaccines.

[CR15] Malemud CJ (2018). Defective T-cell apoptosis and T-regulatory cell dysfunction in rheumatoid arthritis. Cells.

[CR16] Mueller AL, Payandeh Z, Mohammadkhani N, Mubarak SMH, Zakeri A, Alagheband Bahrami A, Brockmueller A, Shakibaei M (2021). Recent advances in understanding the pathogenesis of rheumatoid arthritis: new treatment strategies. Cells.

[CR17] Pozsgay J, Szekanecz Z, Sármay G (2017). Antigen-specific immunotherapies in rheumatic diseases. Nat Rev Rheumatol.

[CR18] Song X, Liang F, Liu N, Luo Y, Xue H, Yuan F, Tan L, Sun Y, Xi C, Xi Y (2009). Construction and characterization of a novel DNA vaccine that is potent antigen-specific tolerizing therapy for experimental arthritis by increasing CD4^+^CD25^+^Treg cells and inducing Th1 to Th2 shift in both cells and cytokines. Vaccine.

[CR19] Song S, Li P, Zhang R, Chen J, Lan J, Lin S, Guo G, Xie Z, Jiang S (2019). Oral vaccine of recombinant *Lactococcus lactis* expressing the VP1 protein of duck hepatitis A virus type 3 induces mucosal and systemic immune responses. Vaccine.

[CR20] Wang L, Wang X, Bi K, Sun X, Yang J, Gu Y, Huang J, Zhan B, Zhu X (2016). Oral vaccination with attenuated *Salmonella typhimurium*-delivered TsPmy DNA vaccine elicits protective immunity against *Trichinella spiralis* in BALB/c mice. PLOS Negl Trop Dis.

[CR21] Wegrzyn G, Wegrzyn A (2002). Stress responses and replication of plasmids in bacterial cells. Microb Cell Fact.

[CR22] Yurina V (2018). Live bacterial vectors-a promising DNA vaccine delivery system. Med Sci.

[CR23] Zeng L, Yang K, Zhang T, Zhu X, Hao W, Chen H, Ge J (2022). Research progress of single-cell transcriptome sequencing in autoimmune diseases and autoinflammatory disease: a review. J Autoimmun.

[CR24] Zhao X, Long J, Liang F, Liu N, Sun Y, Xi Y (2019). Dynamic profiles, biodistribution and integration evaluation after intramuscular/intravenous delivery of a novel therapeutic DNA vaccine encoding chicken type II collagen for rheumatoid arthritis in vaccinated normal rodent. J Nanobiotechnol.

[CR25] Zhao X, Long J, Liang F, Liu N, Sun Y, Xi Y (2019). Vaccination with a novel antigen-specific tolerizing DNA vaccine encoding *CCOL2A1* protects rats from experimental rheumatoid arthritis. Hum Gene Ther.

[CR26] Zhao X, Long J, Liang F, Liu N, Sun Y, Xi Y (2021). Different protective efficacies of a novel antigen-specific DNA vaccine encoding chicken type II collagen via intramuscular, subcutaneous, and intravenous vaccination against experimental rheumatoid arthritis. Biomed Pharmacother.

[CR27] Zheng SY, Yu B, Zhang K, Chen M, Hua YH, Yuan S, Watt RM, Zheng B-J, Yuen K-Y, Huang J-D (2012). Comparative immunological evaluation of recombinant *Salmonella typhimurium* strains expressing model antigens as live oral vaccines. BMC Immunol.

