# Correction to: Transformed *Salmonella typhimurium* SL7207/pcDNA-*CCOL2A1* as an orally administered DNA vaccine

**DOI:** 10.1186/s13568-024-01682-8

**Published:** 2024-03-18

**Authors:** Juan Long, Yang Zeng, Fei Liang, Nan Liu, Yongzhi Xi, Yuying Sun, Xiao Zhao

**Affiliations:** Department of Immunology and National Center for Biomedicine Analysis, Senior Department of Hematology, Fifth Medical Center of Chinese PLA General Hospital, No.8, Dongda Ave, Fengtai District, Beijing, 100071 China

**Correction to: AMB Express (2023) 14:6**

10.1186/s13568-023-01650-8

Following publication of the original article (Long et al. 2024), the author noticed a mistake on the gift information for the attenuated *Salmonella typhimurium* SL7207.

The following are the corrections that need to be made:

1. In the first section of “Materials and methods”, “Frozen attenuated *Salmonella typhimurium* SL7207 (constructed using the original strain [Strain Number: BNCC108207] by Kunming Institute of Zoology, Chinese Academy of Sciences, Kunming, Yunnan, China)” should be corrected to “Frozen attenuated *Salmonella typhimurium* SL7207 (constructed using the original strain [Strain Number: BNCC108207] by Professor Heng Yang, Yunnan Tropical and Subtropical Animal Virus Diseases Laboratory, Yunnan Animal Science and Veterinary Institute, Jindian, Kunming, Yunnan, China) ”.
2. In the section of “Acknowledgments”, “The authors thank the Kunming Institute of Zoology of the Chinese Academy of Sciences for providing the attenuated *Salmonella typhimurium* SL7207 strain used in the present study.” should be corrected to “The authors thank Professor Heng Yang (Yunnan Tropical and Subtropical Animal Virus Diseases Laboratory, Yunnan Animal Science and Veterinary Institute, Jindian, Kunming, Yunnan, China) for providing the attenuated *Salmonella typhimurium* SL7207 strain used in the present study.”
